# Crystal structure of *E. coli* endonuclease V, an essential enzyme for deamination repair

**DOI:** 10.1038/srep12754

**Published:** 2015-08-05

**Authors:** Zhemin Zhang, Qian Jia, Chun Zhou, Wei Xie

**Affiliations:** 1 State Key Laboratory for Biocontrol, School of Life Sciences, The Sun Yat-Sen University, Guangzhou 510275, People’s Republic of China; 2 Center for Cellular & Structural biology, The Sun Yat-Sen University, 132 E. Circle Rd., University City, Guangzhou 510006, People’s Republic of China.; 3 Structural Biology Program, Memorial Sloan-Kettering Cancer Center, 430 E. 67th Street, New York, NY 10065, USA

## Abstract

Endonuclease V (EndoV) is a ubiquitous protein present in all three kingdoms of life, responsible for the specific cleavages at the second phosphodiester bond 3’ to inosine. *E. coli* EndoV (EcEndoV) is the first member discovered in the EndoV family. It is a small protein with a compact gene organization, yet with a wide spectrum of substrate specificities. However, the structural basis of its substrate recognition is not well understood. In this study, we determined the 2.4 Å crystal structure of EcEndoV. The enzyme preserves the general ‘RNase H-like motif’ structure. Two subunits are almost fully resolved in the asymmetric unit, but they are not related by any 2-fold axes. Rather, they establish “head-to-shoulder” contacts with loose interactions between each other. Mutational studies show that mutations that disrupt the association mode of the two subunits also decrease the cleavage efficiencies of the enzyme. Further biochemical studies suggest that EcEndoV is able to bind to single-stranded, undamaged DNA substrates without sequence specificity, and forms two types of complexes in a metal-independent manner, which may explain the wide spectrum of substrate specificities of EcEndoV.

Oxidative DNA damages occur when DNA encounters reactive oxygen species (ROS), which may come from ionizing radiation, chemicals, as well as oxidative phosphorylation. These reactive oxygen radicals greatly promote the spontaneous deamination process of DNA through a hydrolytic reaction^1^,^2^. Deaminated bases by reagents or environmental stresses form common lesions in genome^3^,^4^,^5^,^6^,^7^,^8^,^9^. For example, in nitrosative stresses, adenosine (A), cytidine (C) and guanosine (G) will be deaminated to produce nonstandard bases inosine (I), uridine (U), xanthosine (X) and oxanosine (O), respectively. Due to the strong mismatching abilities of inosine, an A-T base pair will be subsequently converted to a G-C base pair during DNA replication, and this transition potentially leads to mutations in the genome. To counteract the mutational effects of deamination damages, a unique alternative excision repair pathway has evolved, in which the enzyme endonuclease V (EndoV) plays an important role^10^.

EndoV was first discovered in *E. coli* in 1977 and the protein is encoded by the gene *nfi*^11^,^12^. *In vivo* studies revealed that *E. coli* EndoV (EcEndoV) protects cells from mutagenic effects and nitrosative deamination during normal growth^13^,^14^,^15^,^16^,^17^. On the other hand, the *nfi*^–/–^ mice are cancer-prone^18^. EcEndoV primarily incises deoxyinosine (dI) in DNA^19^ at the second phosphodiester bond 3′ to the lesion, producing a nick with a 3′-hydroxyl and a 5′-phosphoryl group^15^,^19^,^20^. In addition, EcEndoV also cleaves the U, O, X, apurinic/apyrimidinic (AP) site, insertion/deletion mismatches (IDM), flaps, loops and pseudo-Y DNA structures, when Mn^2+^ is supplied as a metal cofactor. Studies revealed that *T. Maritima* EndoV (TmEndoV) works via a catalytic and regulatory two-metal mechanism, where Mg^2+^ or Mn^2+^ is responsible for the cleavage while Ca^2+^ stimulates substrate binding^21^,^22^. When only Ca^2+^ is present, EcEndoV forms stable complexes with deoxyinosine-containing DNA, mismatches-containing DNA, flaps, pseudo-Y, fork, 3-way- and holiday junction DNA^23^. In addition, the affinity of EcEndoV towards DNA that contains deoxyinosine is more than 20-fold higher than mismatch-containing DNA, indicating that the 6-keto group of hypoxanthine from deoxyinosine is essential for the stable interactions with the enzyme^24^. Interestingly, the cleavage of mismatch-containing DNA is strand-specific, which can be weakened by an adjacent G-C pair. EcEndoV cleaves the strand whose 5′ terminus is closer to the mismatch. By contrast, it cleaves the deoxyinosine-containing strand exclusively without terminus preference^25^, suggesting that EcEndoV has different cleavage mechanisms against deoxyinosine and mismatches.

Notably, EcEndoV is able to form two types of protein-DNA complexes^24^. When the enzyme concentration is low, one protein molecule binds to one molecule of DNA substrate (complex I). At higher concentrations, a second enzyme molecule binds to complex I and forms a protein-DNA complex in the stoichiometry of 2:1 (complex II). DNase I footprinting assays showed that the first enzyme molecule protects five residues 5′ to the nick site when complex I is formed, while the second enzyme molecule protects at least 13 residues 3′ to the lesion in complex II. In addition, both subunits protect the lesion-containing strand, but no significant protection effects were found for the complementary strand^26^.

Quite a few EndoV structures have been determined since the discovery of EcEndoV^18^,^27^,^28^. Among these, TmEndoV is the most extensively studied enzyme. The protein forms a single α + β domain, composed of a β-sheet of eight mixed strands flanked by α-helices on both sides. TmEndoV contains an ‘RNase H-like motif’, a typical motif in *E. coli* RNase H^29^,^30^, which is also found in the catalytic domain of *E. coli* DNA transposase, 5′ endonuclease domain of the nucleotide excision repair protein UvrC from *T. Maritima*, *E. coli* and yeast Holliday junction resolvase RuvC, and the PIWI domain of *P. furiosus* Argonaute^31^,^32^,^33^,^34^,^35^. TmEndoV with the lesion-containing double-stranded DNA (dsDNA) in different states (2W35, the product state of inosine-containing dsDNA complex; 2W36, the lesion-recognition state of inosine-containing dsDNA complex; 4B20, the loop-containing dsDNA complex) revealed that TmEndoV recognizes the lesions in DNA, and flips the nucleoside ~90° into the lesion-recognition pocket in the opposite direction toward the minor groove. In contrast, other base-excision repair enzymes flip the nucleoside ~180° into the recognition pocket toward the major groove^36^. A conserved wedge motif P79-Y80-I81-P82 (PYIP) of TmEndoV inserts itself into the DNA duplex and separates the two strands near the lesion site. The aromatic ring of Y80 stacks onto the base 3′ adjacent to the hypoxanthine, sterically blocks the empty hypoxanthine position in the DNA helix, and hydrogen bonds with the DNA phosphate backbone. Additionally, P82 and I81 interact with the base 5′ of hypoxanthine and separate the helix. The lesion-recognition pocket is composed of G83, L85, G111, G113, G121 and L142, and the recognition of hypoxanthine only involves the protein backbone^18^. In the product-bound state complex, D43, Q89 and D110 interact with Mg^2+^, and Mg^2+^ is directly coordinated to the 3′ OH group of the 3′ base near hypoxanthine and D43 and D110. On the other hand, K139 and H214 tightly lock the 5′ terminal phosphate of the base adjacent to the nick site.

We have recently solved the crystal structure of human EndoV (hEndoV)^28^, which prefers single-stranded RNA (ssRNA) as substrates to double-stranded RNA (dsRNA) or dsDNA^37^,^38^. Despite the distinct substrate preferences from TmEndoV, the structure of hEndoV-SF resembles that of TmEndoV with an r.m.s. deviation of 2.1 Å over 223 Cα atoms. In addition, the general ‘RNase H-like motif’ is preserved. However, hEndoV has four extra insertion regions compared to the orthologs from lower organisms: insertion 1 (residues A35-P40), insertion 2 (residues K57-S60), insertion 3 (residues Q115-M119), insertion 4 (residues D161-N166) as well as a C-terminal proline-rich loop. Particularly, insertion 4 forms an α-helix, suggesting that this extra region has a certain function. Furthermore, there is a unique four-cysteine motif in hEndoV located at C-terminus and the C-to-S double mutation of the last two cysteines dramatically diminishes the endonuclease activity. However, the detailed mechanism of substrate recognition or cleavage is still lacking and awaits a protein-RNA complex structure for clarification.

EcEndoV is the first member discovered in the EndoV family and it has a broad substrate spectrum. However, no structural studies have been carried out on this intriguing enzyme. To investigate the substrate recognition and catalytic mechanism of EcEndoV, we solved the crystal structure of apo EcEndoV. The 2.4 Å crystal structure of apo EcEndoV and additional biochemical studies shed lights on how EcEndoV carries out the cleavage reaction.

## Results

### Structure overview and comparison to other EndoVs

The full-length version of EcEndoV (EcEndoV-FL) is quite soluble and difficult to be crystallized. Based on the secondary structure analysis as well as results obtained from our limited protease digestion, we discovered a flexible region at the C-terminus. In order to obtain diffraction-quality crystals, the 9-residue, C-terminal fragment was truncated. This truncation reduces protein solubility substantially. The resultant construct is prone to aggregation, but still retains the deoxyinosine-specific endonuclease activity (data not shown). This construct contains residues M1-A214 (named EcEndoV-SF) and it forms plate crystals in a condition containing PEG 3350.

**Figure 1:**
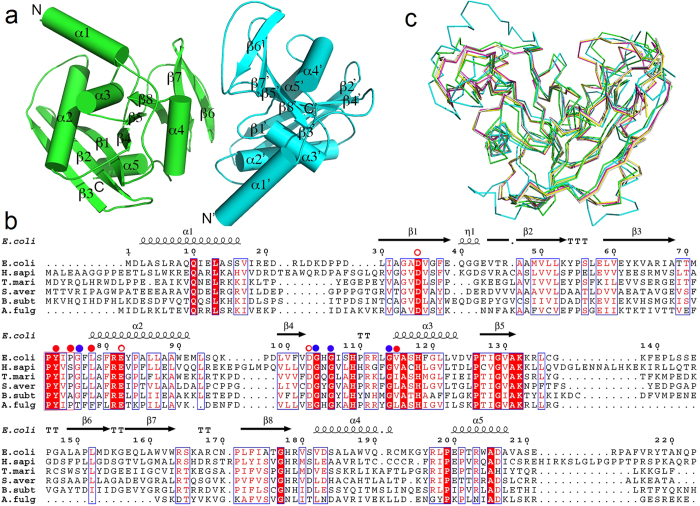
The structural characterization of EcEndoV. (**a**) The overall structure of EcEndoV-SF. The two subunits are colored in cyan and in green. The major secondary elements and the N-, C-termini are labeled. (**b**) Multiple sequence alignment of *E. coli,* *H. sapiens*, *T. maritima,* *S. avermitilis,* *B. subtilis*, *A. fulgidus* EndoV sequences. The full-length EcEndoV sequence is shown and the secondary structure is drawn on the top by ESPRIPT according to the final structure of EcEndoV-SF. The red solid dots represent the hydrophobic residues that form the hypoxanthine-binding pocket, while the blue dots represent the invariant glycine residues that form backbone hydrogen bonds with the base. The open dots are the Mg^2+^-binding residues. Residues identical in all four sequences are highlighted in red while similar residues in blue boxes. (**c**) Structure overlay of Cα traces of EcEndoV-SF (green), hEndoV (cyan, PDB code 4NSP), TmEndoV in the DNA-bound form (magenta, PDB code 2W35), TmEndoV in the DNA-bound form (yellow, PDB code 2W36), TmEndoV in the DNA-bound form (gray, PDB code 4B20). All the DNA components were omitted in the DNA-containing complexes.

The crystals belong to an orthorhombic system with the space group *P*2_1_2_1_2_1_, and each asymmetric unit contains two monomers. Both chains are almost completely intact with no internal disorders and of good electron density: chain A is visible from M1-V208 and chain B is visible from M1-S210. The refinement statistics are summarized in Table 1. The enzyme displays the ‘RNase H-like motif’ (Fig. 1a). Two of the most important elements for substrates binding and recognition are the PYIP motif consisting of residues P72, Y73, I74 and P75, and the hypoxanthine-binding pocket comprising hydrophobic residues Y73, P75, and L78 and V115 (corresponding to I122 in TmEndoV). Additionally, four conserved glycines G76, G104, G106 and G114 also participate in the formation of the rigid pocket and their backbone atoms are involved in hypoxanthine recognition. No Mg^2+^ is found at the cleavage site. Most of these critical residues are conserved in sequence as indicated by the multiple sequence alignment in Fig. 1b.

EndoV is a ubiquitous protein discovered in all three kingdoms of life. A DALI search shows that the closest structural homologues for this protein come from its *T. maritima* ortholog in the free form (PDB code 3HD0), in complex with DNA (PDB code 2W35), *B. subtilis* EndoV (PDB code 3GA2) as well as *S. avermitilis* EndoV (PDB code 3GOC), with decreasing similarity in this order (Fig. 1c). The r.m.s. deviation is 1.4 Å over 204 Cα atoms between EcEndoV-SF and apo TmEndoV. Compared with TmEndoV complexed with DNA, the residues that consist of the PYIP motif of EcEndoV-SF are in similar orientation. Compared to EcEndoV-SF, hEndoV has two extra helices and is also of a larger size. The higher percentage of helical components may have increased structural rigidity, and these variations may confer RNA specificities, suggesting divergent evolutionary paths for the two proteins.

**Figure 2:**
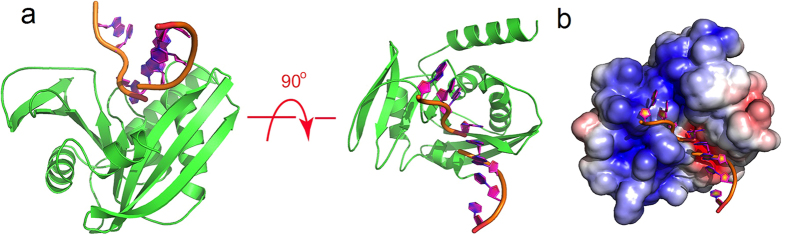
The model of EcEndoV-SF-DNA complex in the stoichiometry of 1:1. (**a**) Two orthogonal views of the complex in the ribbon form, in which the protein and DNA are shown in green and orange, respectively. DNA coordinates were taken from PDB 2W35 and the model was generated by superposition of the protein model of 2W35 onto apo EcEndoV-SF. (**b**) The complex with apo EcEndoV-SF in the surface charge distribution. Surface charge potential is calculated by APBS.

Due to the close structural similarity between EcEndoV-SF and TmEndoV, it is easy to generate a model of EcEndoV-SF complexed with ssDNA substrate. By superposition of apo EcEndoV-SF and TmEndoV in its DNA-bound form, our model shows that ssDNA substrate fits well in the concave face of EcEndoV-SF with the flipped inosine base inserted into the hypoxanthine-binding pocket (Fig. 2a). In addition, the negatively charged DNA molecule neutralizes the positive patches on protein as shown by APBS, making perfect electrostatic complementation (Fig. 2b).

### Asymmetry of the two subunits and interactions between them

**Figure 3:**
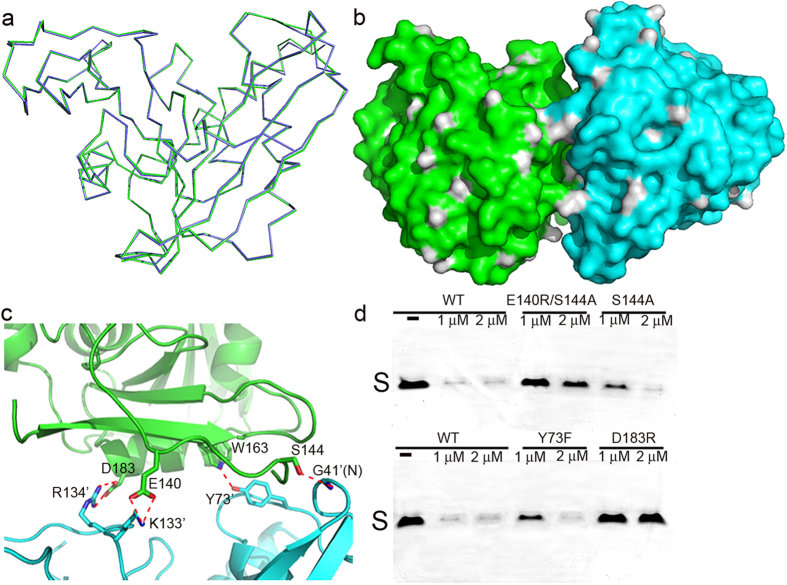
The organization pattern of the two subunits in the asymmetric unit. The color scheme is the same as in Fig. 1a. (**a**) Superposition of chain A backbone onto that of chain B. (**b**) The two subunits shown in surface representation. (**c**) The close-up view of the interactions between the two monomers with the hydrogen bonds indicated by the red dotted lines. (**d**) The cleavage assay of the mutants that disrupt the association mode. The gels were cropped to reduce the size of the figure. Three single mutants S144A, Y73F, D183R and the double mutant E140R/S144A were tested along with WT EcEndoV, with indicated concentrations. The substrate bands are labeled as “S” and the products are too short to be stained by ethidium bromide.

The two monomers within the asymmetric unit are nearly identical although only medium noncrystallographic symmetry (NCS) was implemented during the final stage of the refinement (Fig. 3a). An overlay of their structures shows an r.m.s.deviation of 0.5 Å over 208 Cα atoms. In addition, the two subunits have almost identical B-factors. Interestingly, the two subunits do not form a dimer because there are only very few contacts between them, neither is there a 2-fold axis nor a screw axis to relate one to another. The interfacial area is only 540.9 Å as calculated by the web server PISA and is unlikely to result in the formation of a dimer or higher oligomeric structures. Instead, the two molecules establish “head-to-shoulder” contacts, and the surface rendition of the asymmetric unit is shown in Fig. 3b. Two hydrogen bonds are formed between them: the OG atom from S144 side chain in chain A and G41 backbone nitrogen in chain B, and the NE1 atom of W163 side chain in chain A and Y73 hydroxyl group in chain B. Two additional salt bridges are also found between the carboxylate group of D183 in chain A and the R134 guanidino group in chain B, and the carboxylate group of E140 in chain A and the NZ atom of K133 side chain in chain B (Fig. 3c). However, the side chains of both partners making the D183-R134 salt bridge are highly flexible; consequently this interaction may be weak. Due to the limited contacts, the two monomers do not form a large interface to support a dimer, consistent with our gel filtration chromatography result of a monomer in solution. To find out if the interaction mode between the monomers in the crystal lattice is functionally important, we generated point mutations Y73F, S144A, D183R and E140R. The former two mutants are intended to disrupt the hydrogen bond contacts while the latter two increase the repulsion forces between the two protein subunits within the asymmetric unit. Additionally, the double mutant E140R/S144A has also been designed to test whether any additive or synergistic effects exist between these interactions. With the exception of E140R, which is not expressed in *E. coli*, all the other mutants were well folded in the monomeric form as indicated by their elution profiles on size-exclusion column, and is also supported by dynamic light scattering results (see Supplementary Fig. S1 online). Interestingly, we found that upon mutations, the cleavage capabilities of EcEndoV on dI-containing ssDNA were greatly reduced compared to that of WT (Fig. 3d, see Supplementary Fig. S2 online for the full-length gels).

### EcEndoV is able to bind to nucleic acid substrate without sequence specificity

**Figure 4:**
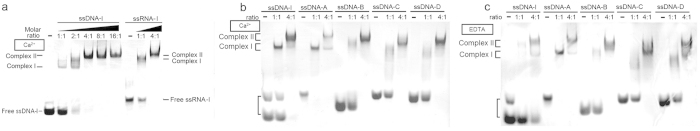
The formation of two types of complexes and the binding behavior of EcEndoV. (**a**) The EMSA of EcEndoV-ssDNA-I/ssRNA-I complexes formed by a constant amount of ssDNA-I, or ssRNA-I (4 μM each) and increasing amounts of protein (molar ratios from 1:1 to 16:1 for ssDNA-I, and 1:1 to 4:1 for ssRNA-I) in the presence of 5 mM Ca^2+^. The two types of complexes and free probes are indicated on each side. (**b**) EMSA of the binding of EcEndoV to nonspecific ssDNA in the presence of 5 mM Ca^2+^. (**c**) EMSA of the binding of EcEndoV to nonspecific ssDNA in the presence of 2.5 mM EDTA.

EcEndoV has been shown to be able to form two types of complexes with DNA in stoichiometry of 1:1 and 2:1 respectively^24^,^26^. Using a 15 nt, single-stranded, inosine-containing DNA or RNA substrate (named ssDNA-I or ssRNA-I respectively), we obtained similar binding patterns in the EMSA assay. EcEndoV starts to form a shift with ssDNA-I or ssRNA-I at a molar ratio of 1:1 in the presence of 5 mM Ca^2+^, which presumably is the complex of EcEndoV bound with one molecule of ssDNA-I/ssRNA-I (complex I). The purpose of Ca^2+^ usage is to inhibit the cleavage activities of EcEndoV^21^,^22^. With increasing amounts of the protein, a supershift (complex II) is also visible. This species dominates in solution at the ratio of 4:1 and higher ratios, at which point very little free ssDNA-I or ssRNA-I is left (Fig. 4a).

Unexpectedly, we found that EcEndoV is able to bind to ssDNA of random sequences. As shown by Fig. 4b, several ssDNA molecules of varying sequences and lengths, form complexes I and II with the enzyme in the presence of 5 mM Ca^2+^. The sizes of these ssDNA range from 15 to 30 nt, and none of them has inosine within its sequence. Furthermore, Ca^2+^ appears to have no effects on the formation of these complexes. We left out Ca^2+^ during the incubation period and also treated the protein with 2.5 mM EDTA to chelate all the possible divalent metal ions, and we still observed the bands of the two complexes with comparable intensities in EMSA (Fig. 4c). This experiment suggested that EcEndoV binds to ssDNA without sequence-specificity, and partially explains the activity of EcEndoV towards multiple substrates, which is consistent with the nonspecific endonuclease and exonuclease activities previously reported in the *E. coli*^20^ and *T. maritima* enzymes^39^,^40^.

## Discussion

In this work we solved the crystal structure of EcEndoV-SF at 2.4 Å and studied its possible evolutionary path from a structural point of view. EcEndoV is a relatively small protein with a compact gene organization. However, it is capable of processing a wide variety of substrates^20^,^25^, the structural basis of which is yet to be investigated. Structural overlay indicates that this protein mostly resembles TmEndoV, which also displays multiple substrate specificities. Modeling studies indicate that EcEndoV binds to the DNA substrate in a similar fashion as TmEndoV. One notable difference is that H214 in TmEndoV is substituted by D210 in EcEndoV. It has been shown that the H214D mutation abolishes the 5′ exonuclease activity in TmEndoV while EcEndoV retains only residual activity^20^,^39^. This observation suggests that the histidine residue plays a critical role in maintaining the exonuclease activity of TmEndoV.

Our 2.4 Å crystal structure of the apo enzyme with two protein molecules in the asymmetric unit reveals an unusual interaction pattern between the two subunits. We eliminated the contacts that connect the two protomers by mutations. These mutants had diminished catalytic abilities, suggesting that the interaction mode between the two subunits may play a functional role and is not just of crystal packing artifacts.

The biophysical and biochemical properties of EcEndoV have been extensively characterized^20^,^23^,^24^,^26^. Previous work largely focused on the cleavage activities of the enzyme on various substrates. These experiments were normally performed under optimal reaction conditions, i.e. in the presence of Mg^2+^ or Mn^2+^, with deoxyinosine-containing DNA duplexes as substrates. Follow-up studies showed that mismatched DNA duplexes, dsDNA with distorted structures, or even undamaged DNA all serve as substrates for EcEndoV^20^,^24^,^26^. EMSA assays carried out by Hitchcock *et al.* demonstrated that TmEndoV binds to dsDNA that contains O in the presence Ca^2+^^41^. Fladeby and co-workers also demonstrated that EcEndoV with Ca^2+^ supplement binds to various dsDNA substrates including undamaged DNA^23^. However, systematic investigation of the binding behaviors of EcEndoV has not been carried out and its binding preferences are still unknown. In this work, we clearly showed that EcEndoV is capable of binding to and forming two types of complexes with ssDNA or ssRNA substrates that contain inosines through EMSA. In addition, we also discovered that EcEndoV binds to random ssDNA sequences even without any divalent metal ions, under which condition the enzyme could behave differently. We also tested ssRNA-I and found that the binding of EcEndoV to ssRNA-I is metal-independent as well (see Supplementary Fig. S3 online).

EcEndoV has about the same affinity for cleaved substrate as the intact substrate. This property allows the second protein molecule to bind to a region 3′ of the cleaved phosphodiester bond as far as 13 bps when in excess, via protein-protein interactions^24^,^26^. This slow turnover process may be attributed to the protective role of EcEndoV to deliver the cleaved product for downstream repair. These interesting phenomena deserve further investigation, and the cocrystal structure of the EcEndoV-ssDNA complex will greatly help us to understand the molecular mechanism underlying the binding behaviors of this enzyme.

## Methods

### Cloning, protein expression and purification of EcEndoV-SF

The wild type, full-length EcEndoV gene (GenBank accession No. YP_491462.1) was amplified from *E. coli* genomic DNA and cloned into pET-28a (+) using the *Nde*I and *Xho*I restriction sites. 19 extra amino acids (MGSSHHHHHHLEVLFQGPH), including a PreScission protease (GE Healthcare Life Sciences) cleavage site, were added to the N-terminus. For crystallization purposes, a truncated fragment of residues M1-A214 (named EcEndoV-SF) was subcloned into pET-28a (+) using the same sites. The mutants were generated by QuikChange method (Stratagene) using EcEndoV-FL as the template. The amplifying and QuikChange primers used in this study were listed in Table 2.

The plasmids encoding the wild type or EcEndoV mutants were transformed into *E. coli* strain BL21 (DE3) cells. The cells were cultured overnight in Luria-Bertani broth containing 50 mg/L kanamycin at 37 °C. 2 L fresh culture medium was then inoculated with 5 mL overnight culture. When OD_600_ reached 0.6–0.8, expression of EcEndoV-SF was induced by 0.3 mM isopropyl β-D-1-thiogalactopyranoside (IPTG) and the culture was kept shaking at 25 °C overnight. The *E. coli* cells were then harvested by centrifugation at 5,000 rpm for 15 min and resuspended in pre-chilled nickel-nitrilotriacetic acid (Ni-NTA) buffer A (20 mM Tris-HCl (pH 8.0), 250 mM NaCl, 10 mM imidazole, 1 mM β-mercaptoethanol and 1 mM phenylmethylsulfonyl fluoride (PMSF)). The cells were disrupted by ultrasonication and the supernatant was obtained by centrifugation at 14,000 rpm for 1 h at 4 °C. The supernatant was then applied onto Ni-NTA affinity resin (Qiagen) equilibrated with Ni-NTA buffer A. The target protein was eluted with Ni-NTA buffer B (20 mM Tris-HCl (pH 8.0), 250 mM NaCl, 250 mM imidazole, 1 mM β-mercaptoethanol and 1 mM PMSF). The EcEndoV-SF fractions were pooled and dialyzed in a buffer consisting of 20 mM Tris-HCl (pH 8.0), 250 mM NaCl, 1 mM PMSF and 1 mM DTT. The dialyzed protein was applied onto a HiTrap Heparin HP column (GE Healthcare) equilibrated with Heparin HP buffer A (20 mM Tris-HCl (pH 8.0), 250 mM NaCl, 1 mM PMSF and 1 mM DTT). EcEndoV-SF protein was eluted in Heparin HP buffer A with increasing NaCl concentration from 250 mM to 1 M. Fractions were dialyzed in Ni-NTA buffer A without imidazole and treated with the PreScission protease overnight (The molar ratio of protease to target protein is 1:150) at 4 °C to remove the N-terminal 6 × histidine tag. The treated protein was loaded onto a Histrap column (GE Healthcare) equilibrated with Histrap column buffer A (20 mM Tris-HCl (pH 8.0), 250 mM NaCl, 1 mM β-mercaptoethanol and 1 mM PMSF). Target protein was eluted with Ni–NTA buffer B. Fractions were concentrated and loaded onto a Superdex 75 size-exclusion column (GE Healthcare) equilibrated with 20 mM Tris-HCl (pH 8.0), 300 mM NaCl, 1 mM DTT and 1 mM PMSF. The final fractions of EcEndoV-SF protein from the size-exclusion column were concentrated to 10 mg/mL using a Millipore centrifugal filter (molecular-weight cutoff 10 kDa) and stored at −80 °C. The wild type full-length EcEndoV and mutants used the same purification protocol as EcEndoV-SF.

### Crystallization and crystallography

Initial crystallization screening was set up by a Mosquito crystallization robot (TTP Labtech) using the sitting-drop vapor-diffusion method with the commercial screens Index (Hampton Research) at 25 °C in 96-well plates. Hits were observed in 2 days and plate crystals were obtained. After optimization, thick plate-shape crystals were obtained from a condition consisting of 26% PEG 3350, 0.05 M sodium fluoride and 0.1 M MES (pH 6.5). Crystals were soaked in a freshly-made cryoprotective solution containing all of the components of the reservoir solution plus 20% (v/v) glycerol. The soaked crystals were mounted on nylon loops and flash-cooled in liquid nitrogen. Using an in-house Oxford Diffraction Xcalibur Nova diffractometer operating at 50 kV and 0.8 mA, a full native data set (a total of 123 frames) was collected with a rotation of 1° per frame at −120 °C. The crystal-to-detector distance was 65 mm and the exposure time was 120 s, and the data were recorded with a 165 mm Onyx CCD detector. The data were processed and scaled using *CrysAlis*^*Pro*^ (v.1.171.33.49; Oxford Diffraction) and *SCALA* from the CCP4 suite^42^.

The structure was solved by molecular replacement using the program *PHENIX*^43^ with the coordinates of hEndoV structure (PDB code 4NSP)^28^ as the search model. There were two molecules in the asymmetric unit. The initial model was extended by *ARP-wARP* using the autobuilding option^44^ and the resulting model was further built manually according to the electron-density map with *COOT*^45^. Multiple cycles of refinement alternating with model rebuilding was carried out by *REFMAC*^46^. The final R-factor was 19.8% (R_free_ = 25.5%) (Table 1). The Ramachandran plot of the final model has 96.14%, 3.86% and 0% of the residues in the most favorable, generously allowed and disallowed region. The final model was validated by *SFCHECK* and *PROCHECK*^47^,^48^. All figures were produced with *PyMOL* (www.pymol.org) and the charge distribution on the protein surface was calculated by APBS^49^. The secondary structure of EcEndoV-SF was prepared by *ESPRIPT* (http://espript.ibcp.fr)^50^. The atomic coordinates and structure factors (code 4XPU) were deposited in the Protein Data Bank, Research Collaboratory for Structural Bioinformatics, Rutgers University, New Brunswick, NJ (http://www.rcsb.org/).

### Electrophoretic mobility shift assays (EMSA)

Single-stranded DNA (ssDNA-I, ssDNA-A, ssDNA-B, ssDNA-C and ssDNA-D) used in our study was chemically synthesized by Life Technologies and their sequences were listed in Table 2. The dry DNA pellet was dissolved in 20 mM Tris-HCl (pH 8.0). The ssRNA-I (5′-CUGAUCGICGAUCAG-3′) was purchased from GE Healthcare and was dissolved in TE buffer (pH 8.0) after deprotection. The standard binding reaction mixture contained 40 mM Tris-OAc (pH 8.5), 5 mM CaCl_2_, 5 mM DTT, 5% glycerol, 4 μM ssDNA or ssRNA and indicated amounts of EcEndoV-FL. The reaction mixture was incubated at 4 °C for 30 min and electrophoresed on a 6% nondenaturing polyacrylamide gel at 4 °C for 70 min. After electrophoresis, the gel was stained with ethidium bromide.

### Dynamic light scattering (DLS) measurements

DLS measurements were carried out with a photogoniometer (plate reader, Wyatt Technology). The protein concentration was set to 40 μM. Samples were subjected to a 10 min-centrifugation at 14,000 rpm to remove large particles. After the samples were loaded on to the plate, the plate was centrifuged at 2,500 rpm for 2 min to remove air bubbles before readout in the temperature-controlled DynaPro plate reader (Wyatt Technology). Each sample was run in triplicates and each well was measured 10 times, with 1 sec acquisition time.

## Additional Information

**How to cite this article**: Zhang, Z. *et al.* Crystal structure of *E. coli* endonuclease V, an essential enzyme for deamination repair. *Sci. Rep.* **5**, 12754; doi: 10.1038/srep12754 (2015).

## Supplementary Material

Supplementary Information

## Figures and Tables

**Table 1:**
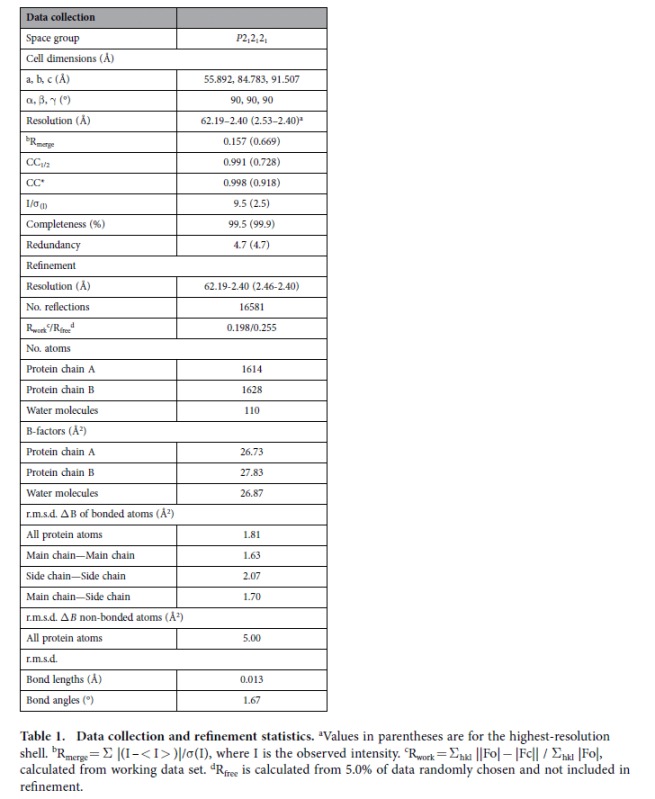
Data collection and refinement statistics.

^a^Values in parentheses are for the highest-resolution shell.

^b^R_merge_ = Σ |(I – < I > )|/σ(I), where I is the observed intensity.

^c^R_work_ = Σ_hkl_ ||Fo| − |Fc|| / Σ_hkl_ |Fo|, calculated from working data set.

^d^R_free_ is calculated from 5.0% of data randomly chosen and not included in refinement.

**Table 2:** The amplifying and QuikChange primers used in this study with mutated amino acids in the sense primers underlined.

Primers	Sense Primers	Antisense Primers
EcEndoV-FL	5′-GATAGGGCCATATGGATCTCGCGTCATTACGCG-3′	5′-TTGCACTTCTCGAGTCAGGGCTGATTTGCTGTATAGC-3′
EcEndoV-SF	5′-GATAGGGCCATATGGATCTCGCGTCATTACGCG-3′	5′-TTGCACTTCTCGAGTCAGGCCACCGCGTCCGCCCAG-3′
EcEndoV-FL-Y73F	5′-TACCACCATGCCTTTCATTCCAGGTTTTC-3′	5′-GAAAACCTGGAATGAAAGGCATGGTGGTA-3
EcEndoV-FL-D183R	5′-CATCGGGTCAGCGTGCGCAGCGCGCTGGCGTG-3′	5′-CACGCCAGCGCGCTGCGCACGCTGACCCGATG-3′
EcEndoV-FL-S144A	5′-CGAACCGCTCTCCGCCGAACCGGGCGCG-3′	5′-CGCGGCCCGGTTCGGCGGAGAGCGGTTCG-3′
EcEndoV-FL-E140R	5′-TATGCGGTAAATTCCGACCGCTCTCCAGCG-3′	5′-CGCTGGAGAGCGGTCGGAATTTACCGCATA-3′
ssDNA-I	5′-CTGATCGICGATCAG-3′	
ssDNA-A	5′-TTCTCCCGCAGCTGC-3′	
ssDNA-B	5′-GTGGTCCATATCCACGCTAG-3′	
ssDNA-C	5′-ACGATTTGCAACTATTCGAACTCCT-3′	
ssDNA-D	5′-CCGGGATCAAGTCCACTCACTTAGATGCAC-3′	
ssRNA-I	5′-CUGAUCGICGAUCAG-3′	
