# Supplementary material for: Crystal structure of E. coli endonuclease V, an essential enzyme for deamination repair
Source: Sci Rep. 2015 Aug 5;5:12754. doi: 10.1038/srep12754 (PMC4650699; doi:10.1038/srep12754)
Supplement: Supplementary Information [file srep12754-s1.pdf]

Supplementary Information for

Crystal structure of *E. coli* endonuclease V, an essential enzyme for deamination repair

Zhemin Zhang[1, 2], Qian Jia[1, 2], Chun Zhou[3], Wei Xie[1,2,*]

[1]State Key Laboratory for Biocontrol, School of Life Sciences, The Sun Yat-Sen University, Guangzhou 510275, People's Republic of China.

[2]Center for Cellular & Structural biology, The Sun Yat-Sen University, 132 E. Circle Rd., University City, Guangzhou 510006, People's Republic of China.

[3]Structural Biology Program, Memorial Sloan-Kettering Cancer Center, 430 E. 67th Street, New York, NY 10065, USA.

[*]To whom correspondence should be addressed.
Tel.: 862039332943; Fax: 862039332847; Email: xiewei6@mail.sysu.edu.cn.

Table of contents

Supplementary Figures S1-3 and Figure Legends

Supplementary Figures S1-3 and Figure Legends

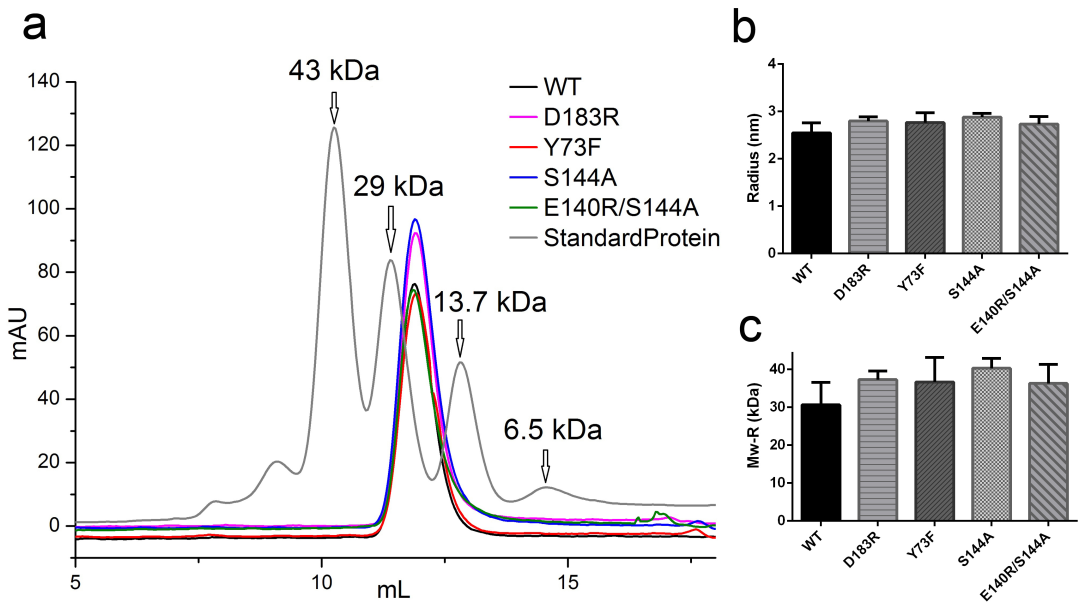

Supplementary Figure S1. EcEndoV is monomeric in solution. (a) The gel filtration chromatograms of EcEndoV and the mutants on a superdex S200 column. The WT enzyme and the mutants are labeled in different colors as indicated by the inset, and the gray line is the trace of four standard proteins with respective molecular masses indicated on the top of each peak. (b) and (c) The results of dynamic light scattering measurements. (b) shows the average particle size of the proteins in solution while (c) gives the estimated molecular weights of each protein. The error bars are calculated from 6 measurements.

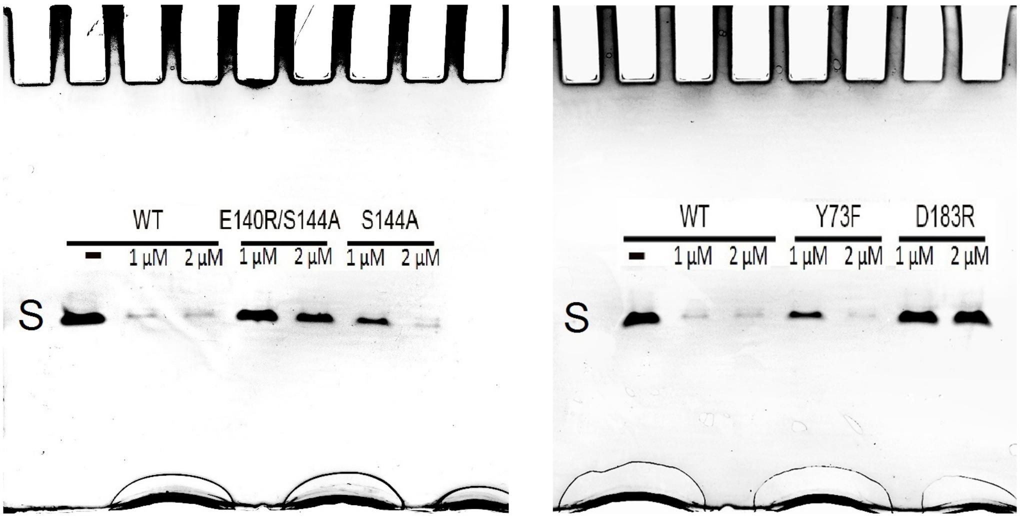

Supplementary Figure S2. The cleavage assay of the mutants that disrupt the association mode (the full-length gel). Three single mutants S144A, Y73F, D183R and the double mutant E140R/S144A were tested along with WT EcEndoV, with indicated concentrations.

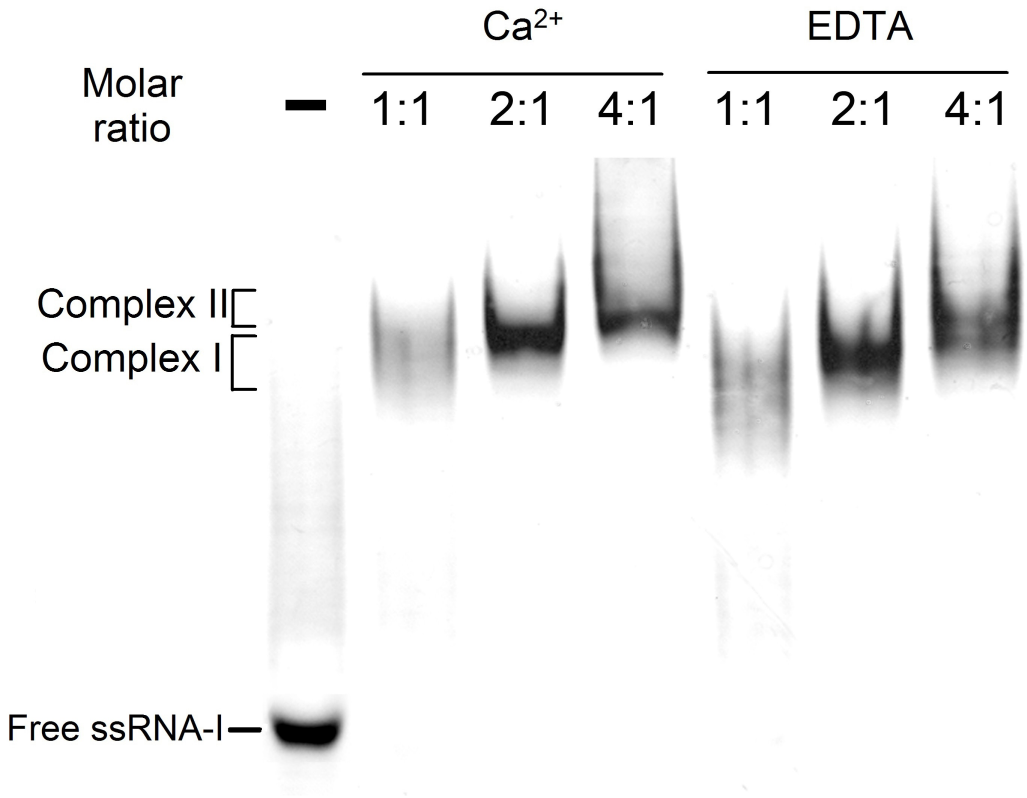

Supplementary Figure S3. The EMSA of EcEndoV binds to ssRNA-I with or without Ca^{2+}. All the lanes contained 4 μM ssRNA-I and the enzyme/RNA ratios are indicated above.
